# Multimodal Evaluation of Body Composition and Muscle Strength in Women Before and After Bariatric Surgery: A Clinical Observational Study

**DOI:** 10.3390/medicina62010182

**Published:** 2026-01-16

**Authors:** Paulo Cesar Grippa, Karina Quesada, Gabriella de Oliveira Barboza, Maria Eduarda Garcia Marvulle, Daniele Candido, Nathália Mendes Machado, Lucas Fornari Laurindo, Adriano Cressoni Araújo, Enzo Pereira de Lima, Elen Landgraf Guiguer, Marcelo Dib Bechara, Cláudia Rucco Penteado Detregiachi, Eduardo Federighi Baisi Chagas, Sandra Maria Barbalho

**Affiliations:** 1 Department of Biochemistry and Pharmacology, School of Medicine, Universidade de Marília (UNIMAR), Marília 17525-902, SP, Brazil; 2 Graduate Program in Structural and Functional Interactions in Rehabilitation, School of Medicine, Universidade de Marília (UNIMAR), Marília 17525-902, SP, Brazil; 3 Department of Biochemistry and Nutrition, School of Food and Technology of Marília (FATEC), Marília 17500-000, SP, Brazil; 4 Laboratory for Systematic Investigations of Diseases, Department of Biochemistry and Pharmacology, School of Medicine, Universidade de Marília (UNIMAR), Marília 17525-902, SP, Brazil

**Keywords:** obesity, sleeve gastrectomy, muscle mass, tomography, muscle strength

## Abstract

*Background and Objectives*: Obesity has been increasing sharply worldwide and is related to diabetes, cardiovascular diseases, liver disease, and cancer. Sleeve gastrectomy is the most used surgical approach to reduce body weight and treat metabolic implications observed in patients with moderate-to-severe obesity. On the other hand, this procedure affects the musculoskeletal system, and investigating skeletal muscle is not routinely recommended for bariatric surgery. This study aimed to evaluate the psoas muscle in patients in the preoperative period of sleeve gastrectomy and six months after the procedure using abdominal computed tomography scans. *Materials and Methods*: This clinical, exploratory, and observational study, with a prospective longitudinal observational study design, was conducted at a single center with 31 women who underwent sleeve gastrectomy. The evaluations were performed before and after six months of the procedures. *Results*: Anthropometric, muscle strength, hepatic ultrasound, and psoas computerized tomography evaluations were performed. A significant reduction in body weight, body mass index, waist, neck, and calf circumference was observed. There was also a substantial reduction in right-hand strength and the area and index of the psoas muscle (but with an increase in density). Most presented a routine abdominal ultrasound. *Conclusions*: Our results suggest that muscle evaluation provides valuable information for clinical monitoring before and after bariatric surgery, helping to identify potential risks and guide multidisciplinary follow-up. Psoas muscle area and psoas muscle index decreased, but psoas muscle density increased, all significantly. These results indicate that conducting a muscle evaluation is helpful for patients undergoing bariatric surgery, supporting the use of the clinical approach before and after the procedure, predicting possible complications, and providing more accurate prognoses.

## 1. Introduction

Obesity is currently closely related to the availability and consumption of high-calorie, nutrient-poor “fast food”, “junk food” or “ultra-processed foods”, as well as calorie-laden soft drinks available at a low cost. In this regard, an unregulated diet reduces the intake of proteins, fiber, vitamins A, C, D, and E, zinc, potassium, magnesium, and calcium, leading to reduced skeletal muscle mass, a reduction potentiated by aging and physical inactivity [1,2]. Thus, the obesity framework is established, inducing inflammation that negatively affects satellite cell function in muscle, increases the likelihood of requiring bariatric surgery, and contributes to the development of sarcopenia [3,4,5]. Moreover, the coexistence of excessive adipose tissue, metabolic dysfunction, insulin resistance, and sarcopenia may lead to higher rates of cardiovascular disease (CVD) occurrence [6,7]. Moreover, obesity is a multifactorial disease also influenced by complex interactions among genetic, hormonal, behavioral, and environmental factors that contribute to its development and progression [8,9,10].

Although obesity affects numerous body systems, including skeletal muscle, evaluating and investigating this system is not routinely recommended for bariatric surgery. Some authors have shown that the clinical picture of a patient undergoing bariatric surgery is consistent with a sarcopenic process [11,12]. Sleeve gastrectomy is the most commonly used surgical approach to reduce body weight and treat metabolic implications observed in patients with moderate-to-severe obesity. On the other hand, this procedure affects the musculoskeletal system [13,14].

Furthermore, the loss of both the quantity and quality of muscle mass significantly impacts health, causing difficulty in wound healing and affecting patient mobilization and glycemic control [15,16]. Also, patients with impaired muscle mass present a higher prevalence of hypercholesterolemia, dyslipidemia, CVD, liver disease, cancer, and cognitive impairment. Obesity is known to increase the risk of these conditions. In the presence of muscle disorders, the risk factors are greatly aggravated. Moreover, inflammatory cytokines secreted by adipose tissue also promote muscle protein breakdown, leading to the loss of fat-free mass [17,18,19,20,21,22,23,24].

Computerized tomography (CT) is widely considered an adequate method for assessing the quantity of muscle or adipose tissue, and it is particularly recommended for evaluating muscle mass [25]. CT is highly recommended for assessing muscle in various processes, including pre- and post-bariatric surgery [12,26,27,28,29,30].

Muscle density is correlated with the degree of fat infiltration, which generally does not interfere with, or only minimally interferes with, the tomographic image. Therefore, CT has the potential to provide more precise and qualitative information about the elasticity or stiffness of soft tissues [31,32]. Thus, CT images allow an estimation of both the quantity (cross-sectional area [CSA]) and quality (radiodensity) of skeletal muscle, visceral adipose tissue, and subcutaneous adipose tissue [33].

Given the importance of assessing the psoas muscle as an indicator of comorbidities, the objective is to evaluate the psoas muscle in patients during the preoperative period of sleeve gastrectomy and at 6 months post-procedure using abdominal CT.

## 2. Materials and Methods

### 2.1. Study Sample

This prospective, longitudinal, observational study was conducted with patients from the High Complexity Service for Individuals with Obesity at the Universidade de Marília (UNIMAR). The sample consisted of 31 women aged 18 to 50 years (pre- and postoperative periods following sleeve gastrectomy), treated at the UNIMAR Charitable Hospital (HBU) in the city of Marília, São Paulo, Brazil. The sample was a convenience sample of patients who completed both pre- and post-surgical assessments during the study period. These 31 women (from a sample of 100) were evaluated at two time points, preoperatively and 6 months postoperatively, with paired comparisons performed for each participant. Only women are treated in the hospital’s bariatric surgery department, where this study was conducted.

This study was approved by the University Ethics Committee (protocol number 7.115.636) and complied with the legal requirements for research involving human subjects established by the Brazilian National Health Council. The patients who agreed to participate in our study signed the Free and Informed Consent Form.

The exclusion criteria were patients with chronic renal failure undergoing dialysis, patients with paresis or hemiparesis due to stroke due to neurological involvement that may influence the results, patients with thyroid diseases due to the role of thyroid hormones in muscle homeostasis, patients with changes in body fluids, such as the presence of edema or ascites regardless of the cause, and patients with difficulty walking. Patients who were chronically using oral corticosteroids were excluded, as were those with any complication requiring surgical intervention, which may necessitate high-protein, high-calorie diets. Patients with the presence of other sequelae of stroke, type 1 diabetes mellitus, or celiac disease; who had a history of malignancy; had undergone a previous bariatric procedure with gastrectomy or colectomy; and weighed more than 200 kg were also excluded. All participants declared right-hand dominance.

### 2.2. Anthropometric Evaluation

The anthropometric data collected included weight and height, and the body mass index (BMI) was analyzed, along with measurements of waist circumference (WC), calf circumference (CC), and neck circumference (NC) to determine metabolic risk. The techniques recommended by Lohman et al., Lohman and Milliken, and Gibson were used. Body weight was collected using a digital platform scale with a maximum capacity of 200 kg (produced by Welmy, Santa Bárbara D’oeste, SP, Brazil). BMI was calculated using the Quetelet formula, in which an individual’s weight (kg) is divided by their height (m) squared [34,35,36,37,38]. These data were assessed following the cutoff points recommended by the World Health Organization. The categorization of metabolic risk based on the WC measurement was carried out following the cutoff points proposed by the World Health Organization, which are normal or no risk (<80 cm for women; and <94 cm for men), high risk (80–87.9 cm for women; and 94–101.9 cm for men) and very high risk (≥88 cm for women; and ≥102 cm for men). The classification of NC measurements was based on a study by Stabe et al. [39], which proposes a cutoff of 39.6 cm in men and 36.1 cm in women, with values above these thresholds associated with greater metabolic risk.

### 2.3. Muscle Strength

Muscle strength was assessed by the grip strength test using a Jamar Hydraulic Hand Dynamometer (Model 5030J1, Patterson Medical, Warrenville, IL, USA). Hand strength assessment is related to an individual’s muscular power and, when reduced, linearly compromises their ability to perform daily activities. A strength of <16 kg for women and <27 kg for men is considered below the expected level, according to the European Consensus on the Diagnosis and Definition of Sarcopenia. For the hand grip strength measurement, the patient was seated without supporting the arms on any backrest. The feet were supported on the floor, and the hips and knees were flexed to 90°. The patient’s shoulders were adducted and in a neutral rotation. The elbow was positioned at 90° of flexion, the forearm was in a neutral position, and the wrist was maintained in a neutral alignment. After positioning the patient, three maximum-grip maneuvers were performed, alternating between the two arms, with at least 1 min of rest between each to control muscle fatigue. The results were expressed in kilograms (kg) as the average of three measurements per hand [32,33,34,35,40]. Handgrip tests were conducted by a trained examiner not involved in CT analysis.

### 2.4. Physical Activity Levels

Physical activity (PA) levels were classified according to the World Health Organization recommendations: light (e.g., walking), moderate (e.g., brisk walking, cycling at a regular pace), and vigorous (e.g., running, aerobic exercise).

### 2.5. Computerized Tomography and Hepatic Ultrasound

CT can distinguish different tissue types based on radiodensity, expressed in Hounsfield units (HU). As the predominant cross-sectional imaging technique, CT can analyze skeletal muscle in various body regions. In addition to muscle area or volume, it is possible to determine the density values of muscles or individual muscle groups. Typically, muscle segmentation and CSA determination are performed at the L3 or L4 level, where the psoas muscle or the entire corresponding area is evaluated. CT is considered the gold standard for assessing body composition and fat distribution due to its high precision and reproducibility and is used for diagnosing numerous post-surgical complications [41,42]. The CT scan procedure followed the work of Kai et al. [13]. All CT analyses were performed by a single experienced radiologist blinded to the time point. Intra-rater reliability was assessed in a subset (n = 10) with an intraclass correlation coefficient (ICC = 0.93). The HU range (−29 to +150) followed validated thresholds.

The patients underwent a single series of abdominal tomography, extending from the diaphragm to the pubic symphysis, without intravenous iodinated contrast, on a multislice CT device (model LightSpeed VCT, GE HealthCare, Milwaukee, WI, USA). The technical parameters used in the exams were the following: collimation of 40 × 1.25 mm, pitch of 1.375:1, thickness of 1.25 mm, KV of 120/140, and mA with dose modulation, ranging from 120 to 600 mA according to the patient’s BMI. For analysis, an image at the level of the L3 vertebra was used; the value of the psoas muscle area (PMA) was obtained through a tool (cross-section) of the after-processing platform AW 4.7 GE. The results of muscle area are often associated with body size to determine the skeletal muscle index (SMI), calculated as: SMI = CSA (cm^2^)/height^2^ (m^2^). In light of the above, it is possible to conclude that a common approach to assess sarcopenia is to quantify the skeletal muscle area (SMA) at the lumbar 3 (L3) level, delineating regions of interest (ROIs) and applying standardized thresholds (−29 HU/+150 HU) to identify muscle tissue, thus obtaining the CSA of the muscle. Commonly used cutoff values for assessing muscle mass in CT scans range from 39 to 41 cm^2^/m^2^ for women and from 52 to 55 cm^2^/m^2^ for men. CT can also be used to assess intermuscular fat. Single-slice CT image analysis can provide a measure of whole-body muscle mass and is minimally affected by the patient’s hydration status. The CSA of skeletal muscles (SMA, cm^2^) at the level of the third lumbar vertebra (L3), normalized by height squared (m^2^), can be used to calculate the SMI (cm^2^/m^2^), which is linearly related to whole-body muscle mass [43,44,45,46].

For the ultrasound evaluations, an ultrasound scanner equipped with a broadband linear transducer probe and an axial resolution of approximately 0.2 mm was used. A preset for system settings was established to improve image acquisition reliability. A radiologist with ultrasound expertise acquired the scanned images after placing the patients in the supine position. The procedures were performed in accordance with other studies [17,47,48].

### 2.6. Statistics

Quantitative variables are described by mean, standard deviation (SD), and 95% confidence interval. To measure the effect of quantitative variables between the pre- and post-moments, the delta (post–pre) was calculated. Qualitative variables were evaluated by absolute (N) and relative (%) frequency distribution. To analyze differences in simple frequency distributions, the Chi-square test for proportions was performed. To analyze the variation in proportion distribution between pre- and post-moment, the McNemar test was performed. To compare the means between the pre- and post-moments, a paired *t*-test was performed after verifying normality with the Shapiro–Wilk test. The Pearson test was used to evaluate the correlation between quantitative variables. The significance level was set at 5%, and the data were analyzed using SPSS (version 27.0). Given the exploratory design and limited sample size, we did not apply formal correction methods to avoid inflating errors.

## 3. Results

Regarding the sample characteristics, the average age was 39.5 years, the average height was 1.62 m, and the average maximum weight was 124.5 kg (Table 1).

The most significant proportion of the sample consisted of mixed-race individuals, married individuals, non-smokers, those with a high school education, and individuals with an income between 2 and 5 minimum wages. Regarding comorbidities, systemic arterial hypertension was the most prevalent. Diabetes and dyslipidemia were present in 19.4% of the sample (Table 2).

During the follow-up period, significant reductions were observed in weight, BMI, WC, NC, and CC (Table 3).

During the follow-up period, a significant reduction in right-hand strength was observed. However, there was no significant effect on left-hand strength and bilateral mean handgrip strength. Considering that the population is right-handed, the main impact is believed to be on the dominant hand (Table 4).

A significant reduction in the area and index of the psoas muscle was observed, accompanied by an increase in density (Table 5).

No significant correlations were found between the delta (Δ) variation in anthropometric and BMI measurements and the Δ variation in hand strength, as well as measurements of the area, index, and density of the psoas muscle (Table 6).

The most significant proportion of the sample underwent a routine abdominal ultrasound, with no significant difference between pre- and post-surgery results. Hepatic steatosis was verified by ultrasound in 29.0% of the sample at the pre-moment and reduced to 16.1% at the post-moment, but without statistical significance (Table 7).

The data indicate that most participants engaged in some form of physical activity (PA), with a high prevalence of walking and moderate-intensity PA. No statistically significant changes in PA behavior were observed between the pre- and post-surgery periods (Table 8).

## 4. Discussion

Basically, bariatric surgery aims to reduce the stomach volume to limit food intake or divert the gastrointestinal tract to limit calorie absorption, leading to weight loss. Sleeve gastrectomy involves the longitudinal resection of the lateral part of the stomach, from the fundus to the antrum, to create a narrow tubular stomach. This procedure promotes weight loss of more than 50%. With the high number of these surgical procedures worldwide, the potential complications also increase, and an accurate, early diagnosis is beneficial for treatment [49,50,51,52,53].

In addition to significant weight loss, bariatric procedures such as vertical sleeve gastrectomy appear to lead to decreases in bone and skeletal muscle mass over time. In this context, procedures termed malabsorptive or mixed result in a more serious musculoskeletal damage due to weight loss, impairment in nutritional absorption, and significant hormonal dysregulations compared to restrictive procedures. This occurs because, after the surgical procedure, mechanical load on the muscle decreases, especially in the first year postoperatively, which is the period of most intense weight loss [44,54,55,56,57,58,59]. When analyzing muscle measurements, it is essential to consider that the benefits include an increased predictive value in clinical research and a more comprehensive screening for low muscle mass. Furthermore, considering that CT-based measurement of skeletal muscle radio-attenuation can also be used to estimate myosteatosis, longitudinal measurements can help identify muscle loss, sarcopenia, and myosteatosis, as well as determine the need for early interventions [60,61].

Although no significant changes in liver ultrasound findings were detected after sleeve gastrectomy, this result remains clinically informative. It likely reflects ultrasound’s limited sensitivity for identifying early or modest reductions in hepatic fat content within a short postoperative period. Furthermore, ultrasound provides a qualitative rather than quantitative assessment of steatosis, which may underestimate subtle improvements that are better detected by CT- or magnetic resonance–based techniques. Therefore, the absence of significant changes does not necessarily indicate the lack of hepatic improvement but rather highlights the need for more sensitive imaging modalities in longitudinal evaluations. 

Furthermore, some correlations between muscle parameters and anthropometric variables did not reach statistical significance; these null findings provide relevant insight into the complexity of postoperative physiological adaptations. The absence of significant associations may reflect the limited sample size, biological variability among participants, or the short follow-up period, during which metabolic and structural changes may not occur synchronously. These results emphasize that muscle mass, strength, and anthropometric variables may follow distinct recovery trajectories after bariatric surgery, underscoring the importance of longitudinal studies with larger cohorts to clarify these interactions.

Kai et al. [13] performed a retrospective study with 86 participants (males and females) who underwent sleeve gastrectomy. Their results were similar to ours regarding the mean patient age (nearly 40 years) and the significant reduction in body weight and BMI after surgery. However, their CT evaluations did not show substantial changes in psoas muscle density (PMD) but did show a significant decrease in the psoas muscle index. Our findings revealed a considerable reduction in psoas area, but a substantial increase in psoas density.

A study by Uno et al. [14] examined the relationships among weight loss, muscle mass, and fat reduction in 29 patients who underwent sleeve gastrectomy. The authors demonstrated that, after a year of surgery, participants lost approximately 31% of their body weight. Moreover, radiological analysis showed a reduction in fat mass and fat mass percentage. They also observed an increase in the percentage of appendicular skeletal muscle mass (22.1% before surgery vs. 28.0% after the procedure). This study concludes that change rates in the percentage of appendicular skeletal muscle mass are positively associated with weight loss and fat reduction.

A study developed by Voican et al. [62] involving 184 obese participants undergoing sleeve gastrectomy exhibited an excellent predictive value for the presence of muscle impairment one year after surgery, where the prevalence of sarcopenia went from 8% at the surgery to 32.0% one year after sleeve gastrectomy. It was also identified that the decrease in muscle mass was significantly correlated with the reduction in body weight as the percentage of initial weight (r = 0.27, *p* < 0.0001) and that sarcopenia is associated with a low initial skeletal muscle mass. In conclusion, the SMA measured by CT scan before and twelve months after sleeve gastrectomy for screening early and late surgical complications is very accurate in evaluating muscle quality and quantity in patients undergoing bariatric surgery.

Our results showed a significant reduction in right-hand muscle strength after 6 months of the procedure. No significant difference was found in the left hand. A prospective cohort study with severely obese patients undergoing preoperative evaluation for bariatric surgery showed a substantial improvement in relation to muscle strength and function after surgery [54]. In a prospective cohort study involving 57 obese patients receiving either protein alone or protein plus branched-chain amino acids (BCAAs) and vitamin D, the authors asked patients to use a hand dynamometer to measure absolute hand grip strength before and 1 month after sleeve gastrectomy. They observed a significant decrease in muscle strength in the protein alone group, which was significantly attenuated in patients receiving combined protein, BCAA, and vitamin D supplementation. The protein, BCAA, and vitamin D supplementation group showed a substantially lower decrease in muscle strength compared to the protein alone group [63]. Handgrip strength, although measured in the upper limbs, is an established proxy for global muscle function and is correlated with total skeletal muscle mass, including lower-limb and trunk musculature such as the psoas.

Regarding the psoas muscle index (PMI), our results showed a significant reduction after the surgery. Rodge et al. [64] evaluated the presence of sarcopenia in cirrhotic patients and showed that this condition was seen in 36%. The authors concluded that the psoas muscle can be used to assess sarcopenia in CT because it is a simple method. Other authors have used the PMI to evaluate this muscle and to increase the use of assertive approaches for several health conditions and surgical procedures [65,66].

In our study, we also investigated hepatic steatosis before and 6 months after surgery. No significant differences were found. Hany et al. [48] investigated the presence of liver disease in patients who underwent sleeve gastrectomy, both before and 6 months after surgery. In their prospective cohort study with 70 obese participants and confirmed metabolic dysfunction-associated fatty liver disease (MAFLD), they observed that the patients also had reduced BMI and improved liver biomarkers. Similar results were obtained by other studies [67,68,69,70,71].

Impaired muscle mass and function are associated with an increased risk of metabolic dysfunction, likely due to the metabolic effects of the patient’s malnutrition before and after surgery. Malnutrition and micronutrient deficiency can lead to the dysfunction of macrophages, neutrophils, and lymphocytes, inhibiting the immune response and augmenting the incidence of postoperative complications. According to some authors, measuring the total psoas muscle using non-enhanced CT is a minimally invasive, accessible tool that can help define muscle quantity, thereby allowing adaptation of patients’ management based on their risk of gastric leak before sleeve gastrectomy. Depending on the results, strategies can be established to favor fat mass loss while maintaining or even increasing lean body mass through hypocaloric diets, protein supplementation, and exercise programs. Furthermore, in some vulnerable cases, preventive strategies may be defined, such as routine drainage, prolonged hospitalization, systematic postoperative CT scan, or even avoiding bariatric surgery [72,73,74,75,76,77].

Many risk factors can trigger muscle mass impairment in obese individuals. Since obesity is characterized by low-grade inflammation and oxidative stress, an augmented release of pro-inflammatory cytokines is associated with insulin resistance, diabetes, hypertension, liver steatosis, cognitive dysfunction, cancer, and other metabolic complications and comorbidities. Furthermore, cycles of weight loss and gain may contribute to reduced skeletal muscle mass. In addition, during these cycles, the regain of fat mass typically occurs without recovery of lean mass [78,79,80,81,82]. Notwithstanding, bariatric surgery may result in a significant decrease in skeletal muscle mass, particularly in the first years after surgery [54,83].

Although most patients in our study reported engaging in some physical activity, there were no significant differences in physical activity levels before and after the surgery. In a prospective study with obese patients undergoing preoperative evaluation for bariatric surgery, 47.8% of patients were sedentary [54]. Physical exercise practice in the preoperative period is highly beneficial for reducing the sarcopenic process, with its success evaluated regarding the intensity, duration, frequency, and progression of physical activity, regardless of age, functional capacity, and the presence or absence of CVD risk factors [84,85]. Furthermore, physical activity contributes to beneficial physiological and metabolic effects that enhance the impact of bariatric surgery on weight loss, including benefits on bone mass, inflammatory markers, endothelial function, and insulin sensitivity [86,87,88,89].

Although the reasoning so far leads us to associate the decrease in muscle mass measured through CT with unfavorable outcomes, this issue should be addressed judiciously because Larsen et al. [90] showed an increased risk of coronary heart disease when there is a greater muscle area, as well as a decrease in this risk when there is a higher abdominal muscle density.

Our study had strength because it was all prospective, used the gold-standard method in terms of muscle evaluation that is CT, a single surgeon performed the procedures and closely followed the evolution of the patients, all data were collected in pre-stated time respecting the pre- and postoperative periods without exceptions to reduce possible biases of patient evolution, and all patients had multidisciplinary team guidance with a unique professional in each specialty, ensuring standardized follow-up and reducing potential bias.

Our study has limitations. First, it included only women. Second, there was a weight limitation due to the CT scanner’s capacity. Additionally, our sample consisted of patients with low financial means, which may have affected their ability to acquire protein supplements. Food surveys that could assess caloric and protein intake were not included, which might have influenced the participants’ muscle condition. Notwithstanding, six months represents an early postoperative stage, which limits assessment of long-term muscle recovery or sarcopenia progression. Finally, a larger sample size, including male participants and a control group, would provide stronger statistical power and broader generalizability. Future studies should address these aspects to confirm our findings. 

## 5. Conclusions 

Our results showed that sleeve gastrectomy is an effective procedure for reducing BMI and anthropometric measures. PMA and PMI decreased, whereas PMD increased significantly. In conclusion, evaluating muscle parameters provides relevant information for clinical monitoring and may help identify early functional and metabolic changes in women undergoing bariatric surgery. These findings highlight the importance of incorporating muscle assessment into postoperative follow-up protocols, rather than indicating a direct therapeutic benefit.

The definition of parameters in tomographic analysis, such as intermuscular adipose tissue (IMAT), low-attenuation muscle (LAM), and normal-attenuation muscle (NAM), should be considered, since area-based evaluations alone may not reflect the predominant tissue composition, potentially leading to misinterpretation. Furthermore, tomographic evaluations gain greater significance when associated with measures of muscle quality. Sarcopenia and sarcopenic obesity are distinct conditions and should have different diagnostic and therapeutic strategies. Therefore, chest and abdominal CT reports should routinely include a description of the muscle condition, as this information may significantly enhance the overall clinical assessment of patients.

## Figures and Tables

**Table 1:** Descriptive data of the mean, standard deviation (SD), and 95% confidence interval (95% CI) for quantitative variables of the sample characteristics (n = 31).

Parameters	Mean	95% CI		SD
		LL	UL	
Age (years)	39.5	36.7	42.3	7.7
Height (m)	1.62	1.60	1.64	0.06
Maximum weight (kg)	124.5	118.4	130.7	16.7

LL: lower limit; UL: upper limit.

**Table 2:** Absolute (N) and relative (%) frequency distribution of the qualitative variables that characterize the sample (n = 31).

		N	%	*p*-Value
Ethnicity	White	11	35.5	0.008 *
	Brown	17	54.8	
	Black	3	9.7	
Marital status	Single	11	35.5	0.008 *
	Married	17	54.8	
	Divorced	3	9.7	
Smoking	Non-smoker	26	83.9	<0.001 *
	Smoker	5	16.1	
Education	Basic	5	16.1	<0.001 *
	High school	17	54.8	
	University	7	22.6	
	Post-graduation	2	6.5	
Income	1 minimum wage	1	3.2	<0.001 *
	1 to 2 minimum wages	8	25.8	
	2 to 5 minimum wages	18	58.1	
	5 to 10 minimum wages	4	12.9	
Diabetes	Yes	6	19.4	0.001 *
	No	25	80.6	
Hypertension	Yes	13	41.9	0.369
	No	18	58.1	
Dyslipidemia	Yes	6	19.4	0.001 *
	No	25	80.6	
Other Diseases	Yes	14	45.2	0.590
	No	17	54.8	

Note: * indicates a significant difference in the distribution of response categories by the Chi-square test for *p*-value ≤ 0.05.

**Table 3:** Comparison of the mean, standard deviation (SD), and 95% confidence interval (95% CI) of anthropometric and BMI measurements between the pre- and post-surgery (n = 31).

Variables	Pre-Surgery				Post-Surgery				*p*-Value
	Mean	SD	95% CI		Mean	SD	95% CI		
			LL	UL			LL	UL	
Body weight (kg)	110.1	14.5	104.9	115.2	87.9	16.0	82.4	93.6	<0.001 *
BMI (kg/m^2^)	42.0	5.6	39.9	43.9	33.5	6.0	31.4	35.7	<0.001 *
WC (cm)	120.1	12.5	115.4	124.6	101.5	2.3	96.3	105.9	<0.001 *
NC (cm)	40.8	3.4	39.5	41.8	37.0	2.4	36.2	37.8	<0.001 *
CC (cm)	47.9	3.9	46.4	49.3	42.8	3.8	41.5	44.2	<0.001 *

Note: * indicates a significant difference between pre- and post-surgery by paired Student’s *t*-test for *p*-value ≤ 0.05. BMI: Body Mass Index (kg/m^2^); CC: Calf Circumference (cm); LL: Lower Limit; NC: Neck Circumference (cm); UL: Upper Limit; WC: Waist Circumference (cm).

**Table 4:** Comparison of the mean, standard deviation (SD), and 95% confidence interval (95% CI) of hand strength measurements between the pre- and post-surgery (n = 31).

Variables	Pre-Surgery				Post-Surgery				*p*-Value
	Mean	SD	95% CI		Mean	SD	95% CI		
			LL	UL			LL	UL	
Right-hand grip strength (kg)	30.6	5.8	28.7	32.6	28.7	5.2	27.1	30.5	0.012 *
Left-hand grip strength (kg)	28.9	6.0	26.9	30.9	28.5	5.0	26.7	30.3	0.556
Bilateral mean handgrip strength (kg)	29.8	5.7	27.9	31.7	28.6	4.8	27.0	30.3	0.054

Note: * indicates a significant difference between pre- and post-surgery by paired Student’s *t*-test for *p*-value ≤ 0.05. LL: Lower limit; UL: Upper limit.

**Table 5:** Comparison of the mean, standard deviation (SD), and 95% confidence interval (95% CI) of measurements of the area, index, and density of the psoas muscle between the pre- and post-moment (n = 31).

Variables	Pre-Surgery				Post-Surgery				*p*-Value
	Mean	SD	95% CI		Mean	SD	95% CI		
			LL	UL			LL	UL	
PMA (cm^2^)	21.9	5.3	19.9	23.7	19.0	4.6	17.4	20.5	<0.001 *
PMI (cm^2^/m^2^)	8.3	2.1	7.6	9.1	7.2	1.8	6.6	7.9	<0.001 *
Mean PMD (HU)	43.9	4.2	42.4	45.3	47.5	4.0	46.0	49.0	<0.001 *

Note: * indicates a significant difference between pre- and post-surgery by paired Student’s *t*-test for *p*-value ≤ 0.05. HU: Hounsfield Units; PMI: Psoas Muscle Index (cm^2^/m^2^); LL: Lower Limit; PMA: Psoas Muscle Area (cm^2^); PMD: Psoas Muscle Density (HU); UL: Upper Limit.

**Table 6:** Correlation analysis of the delta variation in body mass index (BMI) and anthropometric measurements with hand strength and measurements of the area, index, and density of the psoas muscle (n = 31).

	Δ Weight (kg)		Δ BMI (kg/m^2^)		Δ WC (cm)		Δ NC (cm)		Δ CC (cm)	
	r	*p*-Value	r	*p*-Value	r	*p*-Value	r	*p*-Value	r	*p*-Value
Δ RHGS (kg)	−0.077	0.681	−0.149	0.422	−0.254	0.167	−0.131	0.481	0.023	0.903
Δ LHGS (kg)	−0.126	0.501	−0.185	0.319	−0.092	0.621	−0.048	0.800	0.227	0.219
Δ Bilateral Mean HGS (kg)	−0.122	0.512	−0.203	0.274	−0.212	0.252	−0.109	0.558	0.150	0.421
Δ PMA (cm^2^)	−0.192	0.301	−0.242	0.190	0.074	0.693	0.105	0.574	−0.103	0.580
Δ PMI (cm^2^/m^2^)	−0.220	0.234	−0.249	0.176	0.063	0.736	0.064	0.732	−0.147	0.429
Δ Mean PMD (HU)	0.002	0.991	−0.015	0.936	−0.045	0.809	0.218	0.238	0.004	0.985

Note: Pearson’s correlation coefficient (r). *p*-value calculated from Pearson’s correlation test. BMI: Body Mass Index (kg/m^2^); CC: Calf Circumference (cm); HU: Hounsfield Units; RHGS: Right Handgrip Strength (kg); LHGS: Left Handgrip Strength (kg); HGS: Handgrip Strength (kg); NC: Neck Circumference (cm); PMA: Psoas Muscle Area (cm^2^); PMD: Psoas Muscle Density (HU); PMI: Psoas Muscle Index (cm^2^/m^2^); WC: Waist Circumference (cm); Δ: Delta.

**Table 7:** Analysis of the variation in the frequency distribution of abdominal ultrasound assessment and ultrasound to assess the presence of hepatic steatosis between the pre- and post-surgery (n = 31).

Variables		Pre-Surgery		Post-Surgery		*p*-Value
		N	%	N	%	
Abdominal ultrasound	Normal	30	96.8%	31	100.0%	0.999
	Non-routine findings	1	3.2%	0	0	
Liver steatosis (measured by ultrasound)	Yes	9	29.0%	5	16.1%	0.344
	No	22	71.0%	26	83.9%	

Note: *p*-value calculated using the McNemar test.

**Table 8:** Study of the variation in the frequency distribution of physical activity (PA) behavior by type between pre- and post-surgery (n = 31).

Variables		Pre-Surgery		Post-Surgery		*p*-Value
		N	%	N	%	
PA walk	Practiced	24	77.4%	25	80.6%	0.999
	Not practiced	7	22.6%	6	19.4%	
Moderate PA	Practiced	28	90.3%	22	71.0%	0.070
	Not practiced	3	9.7%	9	29.0%	
Vigorous PA	Practiced	9	29.0%	10	32.3%	0.999
	Not practiced	22	71.0%	21	67.7%	

Note: *p*-value calculated using the McNemar test.

## Data Availability

The raw data supporting the conclusions of this article will be made available by the authors upon request.

## References

[1] Zhang Y., Zeng X., Wu F., Yang X., Che T., Zheng Y., Li J., Zhang Y., Zhang X., Wu Z. (2024). Adipocyte-Targeted Nanocomplex with Synergistic Photothermal and Pharmacological Effects for Combating Obesity and Related Metabolic Syndromes. Nanomaterials.

[2] Erol M.F., Kayaoglu H.A. (2024). Comparison of the Effectiveness of Single Anastomosis Sleeve Ileal Bypass and Roux-en-Y Gastric Bypass in Obese Patients with Type 2 Diabetes. Obes. Surg..

[3] Mohapatra S., Gangadharan K., Pitchumoni C.S. (2020). Malnutrition in obesity before and after bariatric surgery. Dis. Mon..

[4] Mancilla-Galindo J., Ortiz-Gomez J.E., Pérez-Nieto O.R., De Jong A., Escarramán-Martínez D., Kammar-García A., Ramírez Mata L.C., Díaz A.M., Guerrero-Gutiérrez M.A. (2024). Preoperative Atelectasis in Patients with Obesity Undergoing Bariatric Surgery: A Cross-Sectional Study. Anesth. Analg..

[5] Hany M., Shafei M.E., Ibrahim M., Agayby A.S.S., Abouelnasr A.A., Aboelsoud M.R., Elmongui E., Torensma B. (2024). The Role of Preoperative Abdominal Ultrasound in the Preparation of Patients Undergoing Primary Metabolic and Bariatric Surgery: A Machine Learning Algorithm on 4418 Patients’ Records. Obes. Surg..

[6] Hussein M., Mirza I., Morsy M., Mostafa A., Hassan C., Masrur M., Bianco F.M., Papasani S., Levitan I., Mahmoud A.M. (2024). Comparison of Adiposomal Lipids between Obese and Non-Obese Individuals. Metabolites.

[7] Mathioudaki A., Fanni G., Eriksson J.W., Pereira M.J. (2024). Metabolomic Profiling of Adipose Tissue in Type 2 Diabetes: Associations with Obesity and Insulin Resistance. Metabolites.

[8] Crispim Carvalho N.N., Baccin Martins V.J., da Nóbrega V.A., de Arruda Neta A., Cavalcante da Fonseca L.A., Bandeira F., de Brito Alves J.L. (2023). Effects of Preoperative Sarcopenia-Related Parameters on Cardiac Autonomic Function in Women with Obesity Following Bariatric Surgery: A One-Year Prospective Study. Nutrients.

[9] Bignotto M., Bianco E., Centofanti L., Russo A., Dei Cas M., Zermiani P., Morano C., Samartin F., Bertolini E., Bifari F. (2024). Synergistic effects of glucose tolerance and BMI on cardiovascular events and all-cause mortality in a healthy population. CA.ME.LI.A study 7 year follow-up. Am. J. Physiol. Endocrinol. Metab..

[10] Ali S.R., Nkembo A., Tipparaju S.M., Ashraf M., Wanling X. (2024). Sarcopenia: Recent Advances for Detection, Progression and Metabolic Alterations along with Therapeutic Targets. Can. J. Physiol. Pharmacol..

[11] Crispim Carvalho N.N., Martins V.J.B., Filho J.M., de Arruda Neta A., Pimenta F.C.F., de Brito Alves J.L. (2023). Effects of preoperative sarcopenia-related parameters on the musculoskeletal and metabolic outcomes after bariatric surgery: A one-year longitudinal study in females. Sci. Rep..

[12] Seo E., Kwon Y., ALRomi A., Eledreesi M., Park S. (2024). A multifaceted and inclusive methodology for the detection of sarcopenia in patients undergoing bariatric surgery: An in-depth analysis of current evidence. Rev. Endocr. Metab. Disord..

[13] Kai K., Fujiwara T., Nagao Y., Oki E., Yoshizumi T., Eto M., Nakashima Y. (2023). Evaluation of bone density and skeletal muscle mass after sleeve gastrectomy using computed tomography method. Bone Rep..

[14] Uno K., Sato K., Watanabe A., Kudo T., Fukushima N., Takahashi K., Masuda T., Kurogochi T., Yuda M., Yano F. (2025). Association of changes in appendicular skeletal muscle mass with weight loss and visceral fat reduction after laparoscopic sleeve gastrectomy. Surg. Today.

[15] Murdock D.J., Wu N., Grimsby J.S., Calle R.A., Donahue S., Glass D.J., Sleeman M.W., Sanchez R.J. (2022). The prevalence of low muscle mass associated with obesity in the USA. Skelet. Muscle.

[16] Bužga M., Pekar M., Uchytil J., Horká V., Malůš J., Vilímek D., Švagera Z., Kutáč P., Holéczy P. (2023). Prevention of sarcopenia in patients with obesity after bariatric and metabolic surgery: The effect of programmed training on the muscle tissue and anthropometric functions—A randomized controlled trial (SarxOb study protocol). Biomol. Biomed..

[17] Ballestri S., Nascimbeni F., Lugari S., Lonardo A., Francica G. (2019). A critical appraisal of the use of ultrasound in hepatic steatosis. Expert Rev. Gastroenterol. Hepatol..

[18] Silveira Rossi J.L., Barbalho S.M., Reverete de Araujo R., Bechara M.D., Sloan K.P., Sloan L.A. (2022). Metabolic syndrome and cardiovascular diseases: Going beyond traditional risk factors. Diabetes Metab. Res. Rev..

[19] Meyer H.J., Dermendzhiev T., Hetz M., Osterhoff G., Kleber C., Denecke T., Henkelmann J., Werdehausen R., Hempel G., Struck M.F. (2024). Body composition parameters in initial CT imaging of mechanically ventilated trauma patients: Single-centre observational study. J. Cachexia Sarcopenia Muscle.

[20] Booranasuksakul U., Tsintzas K., Macdonald I., Stephan B.C., Siervo M. (2024). Application of a New Definition of Sarcopenic Obesity in Middle-Aged and Older Adults and Association with Cognitive Function: Findings from the National Health and Nutrition Examination Survey 1999–2002. Clin. Nutr. ESPEN.

[21] Hsu K.J., Chen S.C., Chien K.Y., Chen C.N. (2024). Muscle Mass Adjusted by Body Height is not Correlated with Mobility of Middle-Aged and Older Adults. Curr. Dev. Nutr..

[22] Deguchi N., Tanaka R., Akita T. (2024). Association Between Sarcopenic Obesity and Frailty Risk in Community-Dwelling Older Women with Locomotive Syndrome: A Cross-Sectional Survey. Cureus.

[23] Chen J., Jia S., Guo C., Fan Z., Yan W., Dong K. (2024). Research Progress on the Effect and Mechanism of Exercise Intervention on Sarcopenia Obesity. Clin. Interv. Aging.

[24] Wang T., Zhou D., Hong Z. (2024). Adipose tissue in older individuals: A contributing factor to sarcopenia. Metab. Clin. Exp..

[25] Yang S., Zhang Z., Chen Q., Hu Y., Su T., Sun X., Jin L. (2024). Correlation between computed tomography-based body composition parameters and hepatic venous pressure gradient in patients with cirrhosis: A systematic review and meta-analysis. Diagn. Interv. Radiol..

[26] Weerink L.B.M., van der Hoorn A., van Leeuwen B.L., de Bock G.H. (2020). Low skeletal muscle mass and postoperative morbidity in surgical oncology: A systematic review and meta-analysis. J. Cachexia Sarcopenia Muscle.

[27] Vogele D., Mueller T., Wolf D., Otto S., Manoj S., Goetz M., Ettrich T.J., Beer M. (2024). Applicability of the CT Radiomics of Skeletal Muscle and Machine Learning for the Detection of Sarcopenia and Prognostic Assessment of Disease Progression in Patients with Gastric and Esophageal Tumors. Diagnostics.

[28] Esposto G., Borriello R., Galasso L., Termite F., Mignini I., Cerrito L., Ainora M.E., Gasbarrini A., Zocco M.A. (2024). Ultrasound Evaluation of Sarcopenia in Patients with Hepatocellular Carcinoma: A Faster and Easier Way to Detect Patients at Risk. Diagnostics.

[29] Khristenko E., Sinitsyn V., Rieden T., Girod P., Kauczor H.U., Mayer P., Klauss M., Lyadov V. (2024). CT-based screening of sarcopenia and its role in cachexia syndrome in pancreatic cancer. PLoS ONE.

[30] Liu Z., Wu Y., Khan A.A., Lun L.U., Wang J., Chen J., Jia N., Zheng S., Xu X. (2024). Deep learning-based radiomics allows for a more accurate assessment of sarcopenia as a prognostic factor in hepatocellular carcinoma. J. Zhejiang Univ. Sci. B.

[31] Nowak S., Kloth C., Theis M., Marinova M., Attenberger U.I., Sprinkart A.M., Luetkens J.A. (2024). Deep learning-based assessment of CT markers of sarcopenia and myosteatosis for outcome assessment in patients with advanced pancreatic cancer after high-intensity focused ultrasound treatment. Eur. Radiol..

[32] Yao L., Petrosyan A., Chaudhari A.J., Lenchik L., Boutin R.D. (2024). Clinical, functional, and opportunistic CT metrics of sarcopenia at the point of imaging care: Analysis of all-cause mortality. Skelet. Radiol..

[33] Furberg H., Bradshaw P.T., Knezevic A., Olsson L., Petruzella S., Stein E., Paris M., Scott J., Akin O., Hakimi A.A. (2024). Skeletal muscle and visceral adipose radiodensities are pre-surgical, non-invasive markers of aggressive kidney cancer. J. Cachexia Sarcopenia Muscle.

[34] Lohman T.G., Hingle M., Going S.B. (2013). Body composition in children. Pediatr. Exerc. Sci..

[35] Lohman T., Milliken L.A. (2019). ACSM’s Body Composition Assessment.

[36] Gibson R.J.P. (1990). Principles of Nutritional Assessment.

[37] Gibson R.S. (2005). Principles of Nutritional Assessment.

[38] Garrow J.S., Webster J. (1985). Quetelet’s index (W/H2) as a measure of fatness. Int. J. Obes..

[39] Stabe C., Vasques A.C., Lima M.M., Tambascia M.A., Pareja J.C., Yamanaka A., Geloneze B. (2013). Neck circumference as a simple tool for identifying the metabolic syndrome and insulin resistance: Results from the Brazilian Metabolic Syndrome Study. Clin. Endocrinol..

[40] Yin Z., Zhou W., Ma J., Chen J., Zhou F. (2024). Arthroscopic dual-bone tunnel repair for palmer type IB injuries of the triangular fibrocartilage complex. BMC Musculoskelet. Disord..

[41] Duprée A., de Heer J., Tichby M., Ghadban T., Mann O., Grupp K., Pinnschmidt H.O., Izbicki J.R., Wolter S. (2021). The value of CT imaging and CRP quotient for detection of postbariatric complications. Langenbecks Arch. Surg..

[42] Guo H., Yang B., Kiryu S., Wang Q., Yu D., Sun Z., Chen Y., Li X., Wang F., Ba X. (2023). Evaluation of the relations between reproduction-related pituitary and ovarian hormones and abdominal fat area-related variables determined with computed tomography in overweight or obese women who have undergone bariatric surgery: A cross-sectional study. Quant. Imaging Med. Surg..

[43] Wang D., Zhang G., Yu Y., Zhang Z. (2024). Imaging of Sarcopenia in Type 2 Diabetes Mellitus. Clin. Interv. Aging.

[44] Holanda N., Crispim N., Carlos I., Moura T., Nóbrega E., Bandeira F. (2022). Musculoskeletal effects of obesity and bariatric surgery—A narrative review. Arch. Endocrinol. Metab..

[45] Vassilev G., Galata C., Finze A., Weiss C., Otto M., Reissfelder C., Blank S. (2022). Sarcopenia after Roux-en-Y Gastric Bypass: Detection by Skeletal Muscle Mass Index vs. Bioelectrical Impedance Analysis. J. Clin. Med..

[46] Arao M., Yajima T. (2024). Computed tomography-based abdominal sarcopenic indices and bio-impedance analysis-based skeletal muscle mass index in hemodialyzed patients. Clin. Nutr. ESPEN.

[47] Park J., Lee J.M., Lee G., Jeon S.K., Joo I. (2022). Quantitative evaluation of hepatic steatosis using advanced imaging techniques: Focusing on new quantitative ultrasound techniques. Korean J. Radiol..

[48] Hany M., Demerdash H.M., Abouelnasr A.A., Torensma B. (2024). Effect of Cytokeratin-18, C-peptide, MHR, and MACK-3 Biomarkers in Metabolic Dysfunction-Associated Fatty Liver Disease After Laparoscopic Sleeve Gastrectomy. Biomark. Insights.

[49] Gagnon C., Schafer A.L. (2018). Bone Health After Bariatric Surgery. J. Bone Miner. Res. Plus.

[50] Ederveen J.C., Nienhuijs S.W., Jol S., Robben S.G.F., Nederend J. (2020). Structured CT reporting improves accuracy in diagnosing internal herniation after laparoscopic Roux-en-Y gastric bypass. Eur. Radiol..

[51] Cornier M.A. (2022). A review of current guidelines for the treatment of obesity. Am. J. Manag. Care.

[52] Froylich D., Pinkhasova D., Borisover E., Gerszman E., Khatib E., Mahamid A., Haddad R., Hazzan D. (2024). Does Patient’s Metabolic and Bariatric Surgery Knowledge Predict Optimal Clinical Outcomes?. Obes. Surg..

[53] Salazar P., Poínhos R., Correia F. (2024). Chrononutrition, eating behaviour, and metabolic health among obese patients elected for bariatric surgery. Chronobiol. Int..

[54] Coral R.V., Bigolin A.V., Machry M.C., Menguer R.K., Pereira-Lima J.C., Contin I., Stock P.V. (2021). Improvement in Muscle Strength and Metabolic Parameters Despite Muscle Mass Loss in the Initial Six Months After Bariatric Surgery. Obes. Surg..

[55] Ruthes E.M.P., Lenardt B.C.C., Lass A.D., Petroski C.A., de Mello M.F., de Andrade Junior A.B., Souza C.J.F., de Matos O., Castelo-Branco C. (2022). Lean mass and strength profile of women submitted to bariatric surgery: Comparison of the EWGSOP2 and FNIH classification for sarcopenia—ASBS program phase II. Gynecol. Endocrinol..

[56] Molero J., Olbeyra R., Flores L., Jiménez A., de Hollanda A., Andreu A., Ibarzabal A., Moizé V., Cañizares S., Balibrea J.M. (2022). Prevalence of low skeletal muscle mass following bariatric surgery. Clin. Nutr. ESPEN.

[57] Baad V.M.A., Bezerra L.R., de Holanda N.C.P., Dos Santos A.C.O., da Silva A.A.M., Bandeira F., Cavalcante T.C.F. (2022). Body Composition, Sarcopenia and Physical Performance After Bariatric Surgery: Differences Between Sleeve Gastrectomy and Roux-En-Y Gastric Bypass. Obes. Surg..

[58] Santos C.A., Cinza A.M., Laranjeira Â., Amaro M., Carvalho M., Bravo J., Martins S., Raimundo A. (2023). A dataset on skeletal muscle mass index, body composition and strength to determinate sarcopenia in bariatric patients. Data Br..

[59] He Y.F., Hu X.D., Liu J.Q., Li H.M., Lu S.F. (2024). Bariatric surgery and diabetes: Current challenges and perspectives. World J. Diabetes.

[60] Li S., Yu H., Zhang P., Tu Y., Xiao Y., Yang D., Bao Y., Han J., Jia W. (2021). The Nonlinear Relationship Between Psoas Cross-sectional Area and BMI: A New Observation and Its Insights into Diabetes Remission After Roux-en-Y Gastric Bypass. Diabetes Care.

[61] Tonnesen P.E., Mercaldo N.D., Tahir I., Dietrich A.W., Amayri W., Graur A., Allaire B., Bouxsein M.L., Samelson E.J., Kiel D.P. (2024). Muscle Reference Values from Thoracic and Abdominal CT for Sarcopenia Assessment: The Framingham Heart Study. Investig. Radiol..

[62] Voican C.S., Lebrun A., Maitre S., Lainas P., Lamouri K., Njike-Nakseu M., Gaillard M., Tranchart H., Balian A., Dagher I. (2018). Predictive score of sarcopenia occurrence one year after bariatric surgery in severely obese patients. PLoS ONE.

[63] Schiavo L., Santella B., Paolini B., Rahimi F., Giglio E., Martinelli B., Boschetti S., Bertolani L., Gennai K., Arolfo S. (2024). Adding Branched-Chain Amino Acids and Vitamin D to Whey Protein Is More Effective than Protein Alone in Preserving Fat Free Mass and Muscle Strength in the First Month after Sleeve Gastrectomy. Nutrients.

[64] Rodge G.A., Goenka U., Jajodia S., Agarwal R., Afzalpurkar S., Roy A., Goenka M.K. (2023). Psoas Muscle Index: A Simple and Reliable Method of Sarcopenia Assessment on Computed Tomography Scan in Chronic Liver Disease and its Impact on Mortality. J. Clin. Exp. Hepatol..

[65] Engelmann S.U., Pickl C., Haas M., Kaelble S., Hartmann V., Firsching M., Lehmann L., Gužvić M., van Rhijn B.W.G., Breyer J. (2023). Body Composition of Patients Undergoing Radical Cystectomy for Bladder Cancer: Sarcopenia, Low Psoas Muscle Index, and Myosteatosis Are Independent Risk Factors for Mortality. Cancers.

[66] Hou X., Hu H., Kong C., Zhang S., Wang W., Lu S. (2024). Psoas attenuation is associated with early postoperative complications in geriatric patients undergoing multilevel lumbar fusion surgery for degenerative lumbar spinal stenosis. BMC Musculoskelet. Disord..

[67] Chow M.D., Otersen K., Wassef A., Kong B., Yamarthy S., Rizzolo D., Yang I., Buckley B., Lu A., Crook N. (2024). Effects of intestine-specific deletion of FGF15 on the development of fatty liver disease with vertical sleeve gastrectomy. Hepatol. Commun..

[68] Shen Y., Zhang B., Hu X., Zhang N., Huang Y., Han T., Sun X., Xiang X., Bi Y., Tang W. (2024). Metabolic surgery results in greater metabolic benefits in patients who achieve healthy weight. Surg. Obes. Relat. Dis. Off. J. Am. Soc. Bariatr. Surg..

[69] Yang C., Zhu D., Liu C., Wang W., He Y., Wang B., Li M. (2024). Lipid metabolic reprogramming mediated by circulating Nrg4 alleviates metabolic dysfunction-associated steatotic liver disease during the early recovery phase after sleeve gastrectomy. BMC Med..

[70] Yu T., Ma X., Cheng Y., Wang Z., Zhang G., Ding H., Yin J., Wang Y., Hu S. (2024). Amelioration of NAFLD by sleeve gastrectomy-triggered hepatocyte regeneration in mice—Experimental research. Int. J. Surg..

[71] Rouhi A.D., Castle R.E., Hoeltzel G.D., Williams N.N., Dumon K.R., Baimas-George M., Wachs M., Nydam T.L., Choudhury R.A. (2024). Sleeve Gastrectomy Reduces the Need for Liver Transplantation in Patients with Obesity and Non-Alcoholic Steatohepatitis: A Predictive Model. Obes. Surg..

[72] Cunningham-Rundles S., McNeeley D.F., Moon A. (2005). Mechanisms of nutrient modulation of the immune response. J. Allergy Clin. Immunol..

[73] Theodorakopoulos C., Jones J., Bannerman E., Greig C.A. (2017). Effectiveness of nutritional and exercise interventions to improve body composition and muscle strength or function in sarcopenic obese older adults: A systematic review. Nutr. Res..

[74] Gaillard M., Tranchart H., Maitre S., Perlemuter G., Lainas P., Dagher I. (2018). Preoperative Detection of Sarcopenic Obesity Helps to Predict the Occurrence of Gastric Leak After Sleeve Gastrectomy. Obes. Surg..

[75] Minawala R., Kim M., Delau O., Ghiasian G., McKenney A.S., Da Luz Moreira A., Chodosh J., McAdams-DeMarco M., Segev D.L., Adhikari S. (2024). Sarcopenia Is a Risk Factor for Postoperative Complications Among Older Adults with Inflammatory Bowel Disease. Inflamm. Bowel Dis..

[76] Han E., Kim M.K., Lee H.W., Ryu S., Kim H.S., Jang B.K., Suh Y. (2024). Myosteatosis predicts bariatric surgery response: A longitudinal study in patients with morbid obesity. J. Clin. Endocrinol. Metab..

[77] Kotek J., Lochman P., Hulek M., Sirovy M., Merkl T., Cermakova E., Kotkova K., Paral J., Dusek T. (2024). Does computed tomography-derived volumometry and densitometry of psoas muscle really correlate with complications in rectal cancer patients after elective surgery?. J. Clin. Imaging Sci..

[78] Lee J., Hong Y.-p., Shin H.J., Lee W. (2016). Associations of sarcopenia and sarcopenic obesity with metabolic syndrome considering both muscle mass and muscle strength. J. Prev. Med. Public Health.

[79] Kreidieh D., Itani L., El Masri D., Tannir H., Citarella R., El Ghoch M. (2018). Association between sarcopenic obesity, type 2 diabetes, and hypertension in overweight and obese treatment-seeking adult women. J. Cardiovasc. Dev. Dis..

[80] Van Aller C., Lara J., Stephan B.C., Donini L.M., Heymsfield S., Katzmarzyk P.T., Wells J.C., Prado C.M., Siervo M. (2019). Sarcopenic obesity and overall mortality: Results from the application of novel models of body composition phenotypes to the National Health and Nutrition Examination Survey 1999–2004. Clin. Nutr..

[81] Tofano R.J., Pescinni-Salzedas L.M., Chagas E.F.B., Detregiachi C.R.P., Guiguer E.L., Araujo A.C., Bechara M.D., Rubira C.J., Barbalho S.M. (2020). Association of Metabolic Syndrome and Hyperferritinemia in Patients at Cardiovascular Risk. Diabetes Metab. Syndr. Obes. Targets Ther..

[82] Biteli P., Barbalho S.M., Detregiachi C.R.P., Dos Santos Haber J.F., Chagas E.F.B. (2021). Dyslipidemia influences the effect of physical exercise on inflammatory markers on obese women in post-menopause: A randomized clinical trial. Exp. Gerontol..

[83] Heymsfield S.B., Gonzalez M.C., Shen W., Redman L., Thomas D. (2014). Weight loss composition is one-fourth fat-free mass: A critical review and critique of this widely cited rule. Obes. Rev..

[84] Vieira F.T., de Oliveira G.S., Gonçalves V.S.S., Neri S.G.R., de Carvalho K.M.B., Dutra E.S. (2022). Effect of physical exercise on muscle strength in adults following bariatric surgery: A systematic review and meta-analysis of different muscle strength assessment tests. PLoS ONE.

[85] Santos C.A., Cinza A.M., Laranjeira Â., Amaro M., Carvalho M., Bravo J., Martins S., Raimundo A. (2023). Effects of physical exercise in sarcopenia on patients undergoing bariatric surgery: A protocol for a randomized clinical trial. MethodsX.

[86] Gil S., Kirwan J.P., Murai I.H., Dantas W.S., Merege-Filho C.A.A., Ghosh S., Shinjo S.K., Pereira R.M.R., Teodoro W.R., Felau S.M. (2021). A randomized clinical trial on the effects of exercise on muscle remodelling following bariatric surgery. J. Cachexia Sarcopenia Muscle.

[87] Cui X., Anatolevna S.T., Wang Y. (2024). Deciphering Blood Flow Restriction Training to Aid Lipid Lowering in Obese College Students through Untargeted Metabolomics. Metabolites.

[88] Brandao C.F.C., Krempf M., Giolo de Carvalho F., Aguesse A., Junqueira-Franco M.V.M., Batitucci G., de Freitas E.C., Noronha N.Y., Rodrigues G.D.S., Junqueira G.P. (2024). Sphingolipid and Trimethylamine-N-Oxide (TMAO) Levels in Women with Obesity after Combined Physical Training. Metabolites.

[89] de Lima E.P., Moretti R.C., Torres Pomini K., Laurindo L.F., Sloan K.P., Sloan L.A., Castro M.V.M., Baldi E., Ferraz B.F.R., de Souza Bastos Mazuqueli Pereira E. (2024). Glycolipid Metabolic Disorders, Metainflammation, Oxidative Stress, and Cardiovascular Diseases: Unraveling Pathways. Biology.

[90] Larsen B., Bellettiere J., Allison M., Ryu R., Tam R.M., McClelland R.L., Miljkovic I., Vella C., Ouyang P., Criqui M. (2024). Associations of Abdominal Muscle Density and Area and Incident Cardiovascular Disease, Coronary Heart Disease, and Stroke: The Multi-Ethnic Study of Atherosclerosis. J. Am. Heart Assoc..

